# Patients and Doctors

**Published:** 1887-12-03

**Authors:** 


### The Doctor's Pulpit.

### PATIENTS AND DOCTORS.

As a regular attendant upon religious services the writer has frequently, as he sat in the pew, felt a very eager desire to have his turn in the pulpit after the preacher had finished and before the congregation had been dismissed. So many things are often said by preachers of all denominations which do not accord with either science or sense, that it is distinctly hard upon a man of strong convictions to hear mere foolish talk addressed to the people, and yet to be compelled by the reverent customs of the day and place to remain perfectly silent. In like manner it is quite certain that many people, particularly men, when they have heard the doctor laying down the absolutely infallible law with pompous effusiveness, would like to tackle the representative of physic and bring him to book with a little wholesome common-sense. Last week, patients were called upon to consider a little homily from the doctor's point of view; it is only fair that this week the doctor should be placed on the form and the patient allowed the free use of the schoolmaster's desk for a brief period. To see ourselves as others see us is generally a profitable experience.

There is a feeling amongst many people that the family doctor is not by any means always so clever as he might be. They hear a good deal said of eminent medical men, specialists and others, and the daily newspapers contribute their quota to the greater glorification of the distinguished few. When they themselves are ill, they naturally wish to have the very best skill brought to bear upon their own case. If the illness be merely trifling, and recovery rapid, they are content with Dr. Mildman; but if there be any hitch or difficulty they look longingly towards Sir Alto Phaeton, and think if they could only have him in attendance their return to health would be prompt and certain. That we contend is not at all unnatural. It may be unpleasant from a doctor's point of view, and unwise as regards the patient's real advantage; but it is exceedingly natural. The patient does not see that the doctor's feelings ought to be of so much weight as his own well-being, neither, indeed, should they. The first necessity in medical practice is that the sick person shall be treated with the best skill of his time, and shall always be restored to health at the earliest possible moment.

It must be admitted that there are family doctors and family doctors. There are men in practice, as we all very well know, whom no doctor would call in when ill himself—careless, dishonest, incompetent men. It is a pity, but it is perfectly true. It is curious, too, to observe how such men are sooner or later correctly valued by their patients. An ordinary man cannot really know anything about medical science or practice; and yet he will often have a shrewd suspicion that Dr. Vacant is a fool, or that Mr. Plausible is a humbug. If such a person has been accidentally saddled with either a fool or a humbug as his medical practitioner, why should he stifle the reasonings of his good sense for the sake of sparing the feelings of a man whom he does not esteem? The medical profession should not be regarded as a safe refuge for the incompetent. The laws of competition have, and ought to have, as full and free play among doctors as elsewhere. Given a man who conscientiously does the utmost that is in him for his patients year after year, and a more or less considerable success generally awaits him. But if the doctor take no interest in his work—merely pursuing it as an escape from a coat out at elbows and an insufficient dinner—is it to be wondered at that his clients are a constantly-diminishing number or that his character is held in lessening regard? In such a case the best thing the patient can do is to give Mr. Idle his *congé* and try elsewhere.

It is not really a difficult thing to be a careful and skilled practitioner. The art of medicine does not demand rare genius for its competent and successful practice. What, however, it does require is the greatest possible degree of honesty, intelligence, and carefulness. It is eminently a matter of details—often of apparently trifling details; and he who cannot habitually condescend to these can never be an accomplished physician. The mere diagnosis and physicking of a patient is a very small part of the business. The sick room, the fire, the window, the bed, the furniture, the food, the drink, the mild amusements, the associations and general surroundings, the family concerns; these and a hundred other circumstances are a necessary part of the doctor's proper regard. If he be a man who takes no interest in, or who cannot be trusted with these, he has missed his calling when he has elected to be a family physician. If he is still young and free it would be well for him to choose a more congenial occupation.

A rather delicate question yet remains to be considered. Many patients complain, and apparently not without reason, that the doctor has a very dull conscience concerning the number of his visits. There are those, not a few, who affirm that they are always afraid to call in the family attendant because when once he comes into the house he cannot be got out again. It is even affirmed that Doctor Tenbairns is so persistently attentive that the only way to get rid of him at all is for the mistress of the house to instruct her servant to say she is "not at home." No doubt these charges are very much exaggerated, just as those are which assert that every lawyer is, *ipso facto*, a man who would sell his soul to the biggest knave in the world for six-and-eightpence. But, whether or no, we feel bound to express the conviction that it is a distinctly immoral act for a doctor to pay professional visits when they are unnecessary. It is an act of the same kind as the trader's who adulterates his goods, or of the gamester's who plays with loaded dice. It is even worse, because the doctor takes advantage of circumstances of grief and anxiety to render services which are not necessary in order to increase his own gains. Many apologies are made for this kind of practice. It is said the doctor must live; that he is always ready to serve his patients at a moment's notice when they require him; and that only by having a sufficient income can he keep his house open and himself in readiness. The most plausible argument of all is that the visiting fee of the ordinary practitioner is inadequate, and not of the same value as the services rendered, and that it is necessary and just to increase the visits in order to restore the balance. With all such reasoning we can by no means agree. When the doctor is called to a patient he is put upon his honour; he is master of the situation, and in such circumstances there can only be one rule of conduct, and that is for him to do exactly the same to the patient and his family as he would wish the patient to do to him if the conditions were reversed.

The temptation, we admit, is great; but it is like all other temptation to wickedness—a thing to be resisted entirely and always. It hardly need be doubted that many practitioners do resist it; vast numbers, indeed, have no time to pay superfluous visits. But every medical man, whatever his inducements to the contrary, should feel bound by every consideration of duty and personal honour to pay just as many visits as the case requires, and no more. Precisely the same motives should regulate the quantity of medicine to be sent in. Of course there are prosperous patients who like far more attendance than is at all necessary. In that case, so long as the doctor explains that he is attending for their satisfaction only, there can be no reason why he should not call half a-dozen times a day if he is desired to do so. All men like to be fairly and honestly treated. A cheat is hateful however his cheating be done. There is nothing the world prizes more than a really honest man. This is specially the case with the world of London. The doctor will find that if he acts with fairness, honesty, and candour towards his patients in every transaction, in a few years his position will be one of the most happy and enviable in the world. In the conduct of a medical practice there is absolutely no need whatever for deception of any kind. There is nothing people appreciate more in medical men than a transparent simplicity of character which, both in the matter of fees and of the daily conduct of any given case, acts with the straightforward honesty of a truthful and unsophisticated child.

